# Orbital angular momentum mode division filtering for photon-phonon coupling

**DOI:** 10.1038/srep40526

**Published:** 2017-01-10

**Authors:** Zhi-Han Zhu, Li-Wen Sheng, Zhi-Wei Lv, Wei-Ming He, Wei Gao

**Affiliations:** 1 Institute of photonics and optical fiber technology, Harbin University of Science and Technology, Harbin 150080, China; 2 National Key Laboratory of Science and Technology on Tunable Laser, Harbin Institute of Technology, Harbin 150001, China

## Abstract

Stimulated Brillouin scattering (SBS), a fundamental nonlinear interaction between light and acoustic waves occurring in any transparency material, has been broadly studied for several decades and gained rapid progress in integrated photonics recently. However, the SBS noise arising from the unwanted coupling between photons and spontaneous non-coherent phonons in media is inevitable. Here, we propose and experimentally demonstrate this obstacle can be overcome via a method called orbital angular momentum mode division filtering. Owing to the introduction of a new distinguishable degree-of-freedom, even extremely weak signals can be discriminated and separated from a strong noise produced in SBS processes. The mechanism demonstrated in this proof-of-principle work provides a practical way for quasi-noise-free photonic-phononic operation, which is still valid in waveguides supporting multi-orthogonal spatial modes, permits more flexibility and robustness for future SBS devices.

One of the strongest nonlinear light-matter interactions occurring in transparency media is photon-phonon coupling, and the interaction is broadly termed as stimulated Brillouin scattering (SBS) in case that the phonons are parametrically generated by optical forces. It has been discovered and widely studied since the invention of the laser, such as the generation of high energy laser pulses, nonlinear optical microscopy, sensors, and optical time delay^1^,^2^,^3^,^4^,^5^. More recently, significant progress in nanofabrication techniques has enhanced our capability to shape and control light-matter interactions, as a consequence, now photon-phonon interactions can be manipulated to an unprecedented degree, ranging from photonic and phononic crystals, chalcogenide and silicon waveguides to dual-nanoweb fiber^6^,^7^,^8^,^9^,^10^,^11^,^12^. These tailorable interactions provide a host of integrated signal processing functions having no all-optical analog^13^,^14^,^15^,^16^,^17^, and in consequence SBS science has been experiencing a revival in recent years.

However, how to suppress the SBS noise arising from the interactions between optical fields and non-coherent phonons (or called thermal phonons) is still a thorny problem^18^,^19^, especially for the case that a large power gap exists between the pump and seed for achieving a high-gain coupling. This is because the particle number of spontaneous phonons, unlike light fields, is associated with materials’ temperature, and the phase-matching between light fields and partial non-coherent phonons is inevitable. It is particularly difficult to filter the noise at the exactly same frequency with the Stokes signal in SBS processes. In this work, we suggest and demonstrate that the SBS noise can be filtered out through adding mode labels to light signals. Owing to the introduction of orbital angular momentum (OAM), a distinguishable degree-of-freedom, even extremely weak signals can be discriminated and separated from a strong SBS noise. This proof-of-principle demonstration shows that in addition to the increase of data capacity, mode division multiplexing can also provide quasi-noise-free operations for future photonic-phononic signal processing devices.

## Results

**Figure 1:**
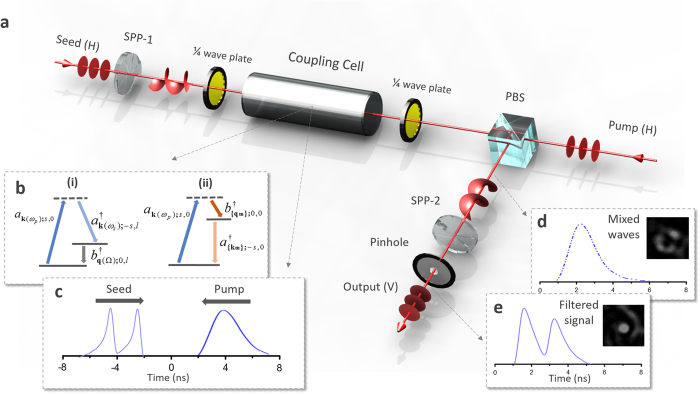
Schematic illustration of OAM mode division filtering for photon-phonon coupling. (**a**) Experimental setup. Key components include Brillouin coupling cell, spiral phase plate (SPP), polarized beam splitter (PBS); 1/4 wave plate, and pinhole. (**b**) Energy level diagram for SBA (i) and SBS-noise generation process (ii). (**c**) Time-domain waveforms of input seed and pump pulses. (**d**) Time-domain envelop and intensity distribution of the output mixed waves when a LG_01_ seed at 10^−8^ J level is injected. (**e**) Time-domain waveform and intensity distribution of the amplified LG_01_ seed (10^−8^ J level) filtered from strong SBS noise (SNR below 5%).

OAM is a fundamental degree-of-freedom of waves, involving light and matter waves, and its paraxial eigenstates, Laguerre-Gauss (LG) modes, form an infinite dimension Hilbert Space. Because of this unique profile, it has gained a rapid development in a wide range of areas, and especially in informatics^20^,^21^,^22^,^23^,^24^,^25^,^26^,^27^,^28^,^29^,^30^. It is worth noting that although mechanical waves, or rather phonons, carry no spin angular momentum (SAM) due to its physical nature of electrostatic oscillations, they can carry OAM by forming a vortex phase^31^. More interesting, our recent work on this front has shown the feasibility and potential of using OAM multiplexing in photonic-phononic signal processing^32^,^33^. Here, to demonstrate its ability of SBS noise filtering, a backward stimulated Brillouin amplification (SBA) with strong pumps and weak OAM seeds is used, in which the intensity of SBS noise is usually much larger than the amplified seed. In SBA, counterpropagating pump and seed waves of frequency *ω*_*p*_ and *ω*_*s*_ interact via coherent generation of an idler acoustic wave with Brillouin frequency Ω = *ω*_*p*_ − *ω*_*s*_. The phase-matching condition in SBA is **q**(Ω) = **k**(*ω*_*p*_) − **k**(*ω*_*s*_), where **k**(*ω*) and **q**(Ω) are the optical and acoustic dispersion relations, respectively. Notice that the momentum conservation required by the phase matching includes SAM and OAM as well, and a LG boson particle can be described as a creation operator 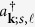 acting on a vacuum state, i.e., 
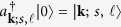
. Thus, the total momentum conservation of SBA interaction, as shown in Fig. 1(b) (i), can be expressed as


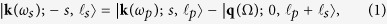


where 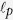, 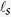, and 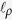 are azimuthal indices of pump, seed, and phonon, respectively, *s* represents SAM, and *s* = ±1, 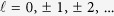. Note that SAM of phonons should always be 0 and OAM of parametrically excited phonons depends on both pump and seed states, and the seed’s momentum keeps constant in SBA, i.e., 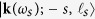
33. Besides SBA, there is another Stokes-type interaction in SBS, where the pump directly interacts with acoustic waves and generates an idler Stokes optical waves, and once the interaction involves spontaneous non-coherence phonons, ASE-like SBS noise will be produced. The phase-matching condition of this noise generation is {**k**_*m*_} = **k(***ω*_*p*_) − {**q**_*m*_}, where the sets of {**q**_*m*_} and {**k**_*m*_} correspond to non-coherent phonons and SBS noise, respectively. According to the fluctuation-dissipation relation (FDR) and the requirement of system OAM conservation, the thermal phonons of a system in thermal equilibrium can be expressed in LG space as 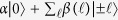, where 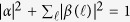. In an ideal homogeneous and isotropic material, the distribution of the thermal phonons largely depends on the geometric size and boundary condition of waveguide^34^,^35^,^36^. For a bulk isotropic medium used in this work—no boundary condition affording axis of rotational symmetry, any edge or screw dislocation motions will be dragged (or damped) by the thermal motion of a uniform distributed lattice. Namely, most of the thermal phonons populate in the fundamental mode of LG space, i.e., |*α*|^2^ ≈ 1. It should be note that even absorbing a torque from outside to generate a vortices, similar as other coherent traveling phonons, this ordered motion will also be dissipated with an exponential decay upon the lifetime of coherent phonons^31^,^33^. Moreover, the SBS-noise generation process (a kind of external perturbation) only reinforces the amplitude of local vibration modes without changing its transverse spatial distribution. Thus the total momentum conservation of SBS-noise generation shown in Fig. 1(b) (ii) can be approximately expressed as


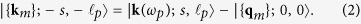


Photon-phonon couplings described in Eq. (1) and Eq. (2) all belong to SBS processes, i.e., a positive feedback loop leads to an energy flow from pump to Stokes and phonon waves, the only difference is that the SBA originates from Stokes seed and the other from thermal phonon noise. The momentum conversion relations shown in Eq. (1) and (2) indicate that OAM degree-of-freedom provides an interface to discriminate the seed from the SBS noise. More specifically, if a Gaussian pump 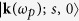 is used in SBA, the amplified OAM seed and SBS noise should be 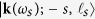 and 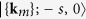, respectively, and therefore the seed can be “de-multiplexed” from noise background via mode conversion and spatial filtering. Moreover, it should be noted that system SAM conservation in backward SBS geometry requires pump and seed in opposite circular or same linear polarization.

Considering the fact that there is no appropriate OAM waveguides so far to perform high efficient photon-phonon coupling, hence a backward SBS in bulk media is used for demonstrating this principle. The experimental setup is illustrated in Fig. 1(a), an H-polarized 532 nm Gaussian pulse with pulse-width 3.5 ns is converted into right-circular polarization by a 1/4 wave plate, and then directed into the coupling cell as the pump wave. On the other side of the coupling cell, double 1.5 ns Gaussian pulses (an easily recognizable waveform) with a Stokes-frequency shift are used as the seed wave, and a spiral phase plate (SPP-1) and 1/4 wave plate are employed to converted it into left-circular polarized LG modes (*ℓ* = 1 or *ℓ* = 2) before entering into the cell. For a sufficient SBS-noise generation, a Brillouin active liquid CS_2_ is chosen as the nonlinear medium injected into the coupling cell. Once a weak seed signal (double pulses at 10^−4^–10^−13^ J level) interacts with a strong pump (mJ level) in the medium, besides partial pump photons will be coherently converted into seed photons and idler phonons, as shown in Fig. 1(b) (i); most of the pump photons will directly couple with non-coherent phonons due to a large energy gap between the pump and seed pulses in this experiment, as shown in Fig. 1(b) (ii), where a strong ASE-like SBS noise will be simultaneously produced. The mixed waves of amplified seed and SBS noise outputting from right side of the cell is converted into V-polarized by a 1/4 wave plate and reflected from the PBS. Then, the amplified seed (mixed in SBS noise) is collimated by a 4 f system (not depicted in Fig. 1(a)), and another spiral phase plate (SPP-2) and an iris diaphragm (pinhole) are employed to “de-multiplex” the seed signal from the strong SBS noise. The amplified seed pulses filtered from SBS noise are detected by an energy meter and photodetector, and a CCD is employed to monitor and record the intensity distribution of the output waves. Figure 1(d) shows a typically local intensity distribution and time-domain waveform of the output waves, when a LG_01_ mode labeled seed at 10^−8^ J level is injected, before OAM mode filtering. It can be seen that the seed signal is completely submerged in the SBS noise with a 2 ns Gaussian-shape envelop. However, as shown in Fig. 1(e), the amplified seed signal can be discriminated and separated from the strong noise after mode filtering.

**Figure 2:**
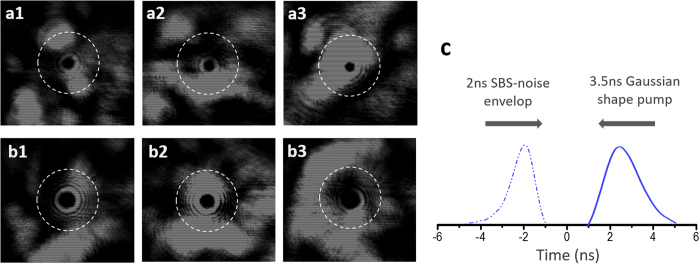
Spatial and temporal properties of ASE-like SBS noise generated from a bulk medium. (**a,b**) Observed beam profiles at different moments of the ASE-like SBS noise projected on LG_01_ mode (a1–a3) and LG_02_ mode (b1–b3), the diameter of the dashed circle is 4 mm. (**c**) Time-domain waveforms of the input pump and output SBS-noise pulses.

**Figure 3:**
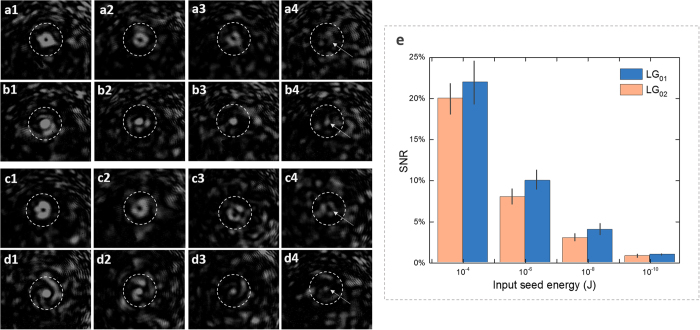
Experimental results of the output waves. (**a–d**) Observed intensity distributions of the output waves as the energy level of the input seed is 10^−4^, 10^−6^, 10^−8^, and 10^−10^ J, respectively, before (a1–a4) and after (b1–b4) mode conversion in specific case of LG_01_ mode employed, and analogous data in specific case of LG_02_ mode employed (c1–c4 and d1–d4), the diameter of the dashed circle is 4 mm. (**e**) Measured signal to noise ratio (SNR) of the amplified seed signals in SBS-noise background versus the level of the input seed energy, where the energy of the mixed output waves is 0.8 mJ-level all the time.

We first analyze the spatial and temporal properties of ASE-like SBS noise generated in this experimental configuration. Here, the seed signal is blocked and strong pump pulses (3 mJ@3.5 ns) are injected into the coupling cell alone to excite SBS noise. Then the output noise is projected onto LG_01_ and LG_02_ modes, respectively, to analyze its energy distribution in corresponding OAM channels. It can be seen that the SBS-noise is a 2 ns pulse envelop at 0.8 mJ level as shown in Fig. 2(c). Figure 2(a1–a3) and (b1–b3) show the intensity profiles of the noise projected onto LG_01_ and LG_02_ modes at different moments, respectively. It can be seen that they are all random speckles inlaid with a pure hollow and no energy readings above environmental noise (50 pJ-level) can be observed in the hollow area. Furthermore, the conversion efficiency of the SPP used here is better than 95% for *l* = 2@532 nm and the total energy of the SBS noise generated here is 0.8 mJ-level. Therefore, the SBS noise generated here can be approximately seen as no vortices as discussed above, and the experimental results shown in Fig 3. (a weak signal less than 5% of the total energy can be filtered out) also confirm this point. However, it should be noted that the geometric boundary condition of waveguides will remarkably change this situation, for instance, cylinder waveguide will increase the energy proportion of high-order mode in LG space^34^. In addition to the geometric boundary condition, microstructure in anisotropy media will also remarkably shape and modulate the spontaneous vibration modes and even a coherent traveling-waves excited from outside^36^.

Next we experimentally demonstrate the feasibility and performance of the OAM mode division filtering, the pump energy is fixed at 3 mJ and the seed energy is set in a range of 10^−4^ to10^−10^ J. The pump and seed pulses are directed into the coupling cell simultaneously for sufficient SBS-noise generation. Figures 3(a1–a4) and (c1–c4) illustrate the intensity distributions of the output mixed waves in specific cases of mode LG_01_ and LG_02_ labeled, respectively. It can be seen that the amplified OAM seed signals are covered (interference with the noise-OAM patterns shown in Fig. 2) by ASE-like SBS noise and completely overwhelmed with the decrease of seed energy. Here, just as the pure SBS noise shown in Fig. 2(c), the waveform of the mixed waves are still a 2 ns Gaussian-shape envelop all the time as shown in Fig. 1(d), and the waveform of the amplified seed is also submerged. Furthermore, the total energy of the output mixed waves is still at 0.8 mJ level, same as the situation of pure SBS-noise generation discussed above. This indicates that noise generation is a predominant process in this configuration, which is consistent with the experiment designed. Figures 3(b1–b4) and (d1–d4) show the corresponding intensity distributions of the output waves that experience an inverse mode conversion, where the OAM labeled signals are converted back to Gaussian shape and the noise are squeezed out from the center region. In consequence, as shown in Fig. 3(b4,d4), even extremely weak signals can be spatially separated from the strong background noise, and the double-pulses waveform of the amplified seed signal become clearly visible via adjusting the aperture of the iris diaphragm (pinhole) as shown in Fig. 1(e). Moreover, the separation effect of mode LG_02_ is better than that of LG_01_, or rather the double-pulse waveform can completely emerge from the noise waveform (as shown in Fig-1(d)) when a bigger pinhole’s aperture is employed, since the Gaussian patterns extracted from SBS noise with LG_02_ mode are cleaner and rounder. This is ascribed to the radius of LG beams’ maximum intensity, 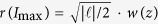, increasing with the azimuthal index 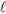 of LG modes^37^, and thus a high-order LG mode can be employed on-demand to achieve a better signal-noise separation. The energy ratio, or to say signal to noise ratio (SNR), between the amplified seed signal and SBS noise in the mixed waves is illustrated in Fig. 3(e), and in the experiment, the total energy of the mixed waves is 0.8 mJ-level all the time due to the predominance of SBS-noise generation. With decreasing the input seed energy, the SNR decreases continuously and reduces to about 1% as the seed energy drops to 10^−10^ J level, which means a weak signal only accounting for 1% of the total output energy can be discriminated. Moreover, the SNR of LG_02_ mode is lower (or to say exhibiting a better filtering ability) than that of LG_01_ mode at the same noise background, this can be attributed to two reasons: firstly, due to a bigger beam size, the energy density of the LG_02_ mode is lower, which leads to a weaker amplified signal contained in the output mixed waves; secondly, this weaker signal can be well discriminated by using LG_02_ mode which has a better ability of signal-noise separation. About this SNR, one may doubt that, as discussed above, the most propagating directions of the SBS noise are different from the amplified seed wave. However, it should be noted that the intensity distributions are observed in free space in this proof-of-principle experiment. In contrast, the signal and noise will overlap in the same space in case that the interaction occurs in waveguides, and this mechanism will be still valid because the signals are labeled with a distinguishable degree-of-freedom.

**Figure 4:**
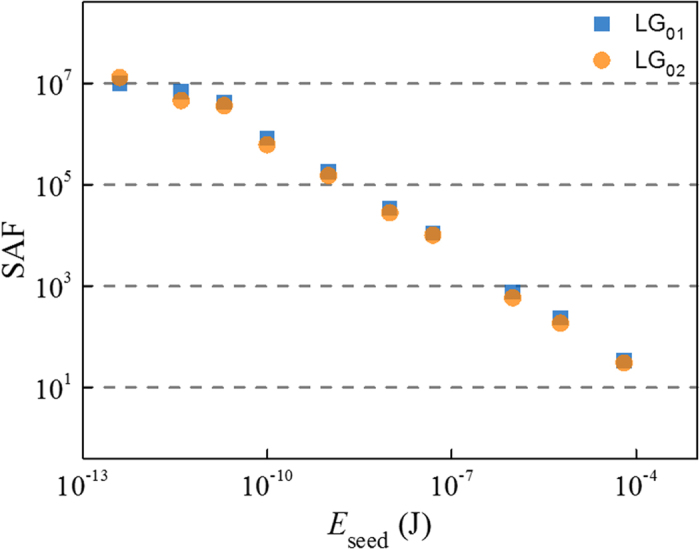
Signal amplification factor (SAF) versus the seed energy *E*_seed_.

In high-energy field, due to the properties of high-efficient energy transfer and elements damage-free, SBA has been proposed and used in Cross-Beam Energy Transfer scheme at the National Ignition Facility^1^. In integrated photonics domain, SBA is also a key component for many SBS-based devices^16^, in which current focus is mainly on enhancing the coupling efficiency. However, for pursuing a high amplification ratio with short interaction length (or to say high-gain coupling), a larger power gap between the pump and seed is necessary, and in consequence the generation of SBS noise is inevitable in room temperature. Moreover, although non-collinear configuration can separate the seed and noise automatically^32^, this configuration only apply to beam combining system used in high-energy domain where a high efficiency of energy transfer is more preferred. To address this dilemma, we also demonstrate a quasi-noise-free strong SBA with short interaction length via OAM mode filtering. Here, the pump energy is set at 4 mJ, and a 3 ns seed is reduced from 10^−4^ J to 10^−13^ J with mode LG_02_ labeled. A diagram of signal amplification factor (SAF, the ratio of the output energy to the input energy) versus the input seed energy is illustrated in Fig. 4, where the SAF increases linearly with the decrease of input seed energy, and a SAF of 10^7^ is obtained as the seed is reduced by about 10^−13^ J. This result suggests that the OAM mode division filtering is a practical solution for designing a quasi-noise-free Brillouin amplifier. It is worth to note, besides the proposed chip-level OAM photonic-phononic waveguide, this mechanism can be used in OAM fiber^38^ to realize a quasi-noise-free Brillouin fiber amplifier. It should be noted that the design of the OAM fiber can modulate the transverse-spatial property of the phonon field remarkably, more important, the phase-matching condition of photon-phonon coupling is also very different from normal fibers due to a unique mode dispersion, and this will be discussed in the future.

## Discussion

In conclusion, we have proposed and demonstrated OAM mode division filtering for photon-phonon coupling. Through a bulk medium SBS, we have shown that even an extremely weak signal can be discriminated and separated from a strong background SBS noise. In addition, by employing OAM mode filtering, a quasi-noise-free strong SBA is achieved in a short interaction length. These results suggest that OAM multiplexing is promising way to enhance photonic-phononic signal processing technique, not only significantly boosting data capacity but also permitting a quasi-noise-free operation for future SBS devices. Although the research on OAM waveguides is still in a very initial stage, a quasi-noise-free Brillouin fiber amplifier based on OAM fiber can be achieved in the near future. Furthermore, this mechanism may work in other light-matter interaction schemes, where noise arises from the coupling between light and non-coherent matter waves, such as atom, plasma, electron, and magnons.

## Method

In the experiments, A frequency-doubled Q-switched Nd:YAG laser produces single-longitudinal and single-transversal Gaussian pulses with beam diameter 1.5 mm at 3.5 ns duration and linear polarization at 1 Hz repetition rate. The 1.5–3 ns Stokes seed is generated via focused-SBS with the nonlinear medium of CS_2_, and the double-pulses signal is produced by an unequal M-Z interferometer path. The length of the coupling cell is 60 cm. The OAM beams in this work are converted by SPPs (RPC Photonics, VPP-1c and Vpp-1064) from Gaussian beam. Different energy detectors (Ophir PE-9, PD-10 and Newport 818E-10-25-S) are used, depending on laser energy level, and the temporal waveform is detected by a 10 GHz-bandwidth Ophir photodetector linked with a 10 GHz-bandwidth oscilloscope.

## Additional Information

**How to cite this article**: Zhu, Z.-H. *et al*. Orbital angular momentum mode division filtering for photon-phonon coupling. *Sci. Rep.* **7**, 40526; doi: 10.1038/srep40526 (2017).

**Publisher's note:** Springer Nature remains neutral with regard to jurisdictional claims in published maps and institutional affiliations.
